# Supplementary material for: Generation of antigen-specific mature T cells from RAG1−/−RAG2−/−B2M−/− stem cells by engineering their microenvironment
Source: Nat Biomed Eng. Author manuscript; Available in PMC 2024 May 12. (PMC11087257; doi:10.1038/s41551-023-01146-7)
Supplement: Chang et al, NBME 2023 Supplemental figs [file NIHMS1956328-supplement-Chang_et_al__NBME_2023_Supplemental_figs.pdf]

nature biomedical engineering

Article

https://doi.org/10.1038/s41551-023-01146-7

Generation of antigen-specific mature T cells from *RAG1*$^{-/-}$*RAG2*$^{-/-}$*B2M*$^{-/-}$ stem cells by engineering their microenvironment

In the format provided by the authors and unedited

Supplementary Figure 1

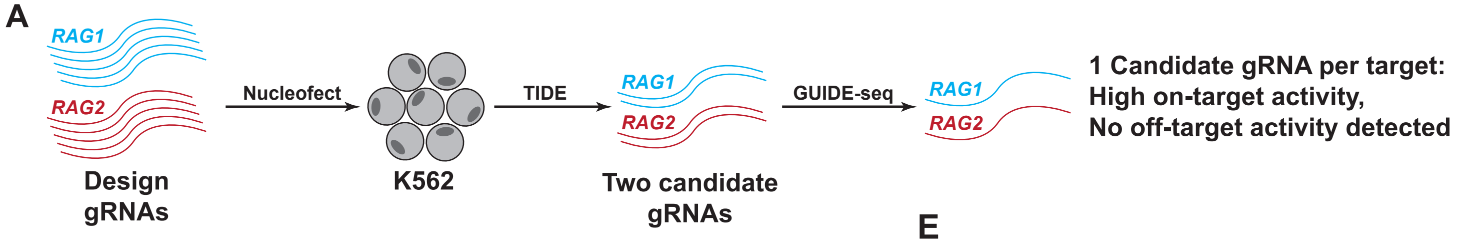

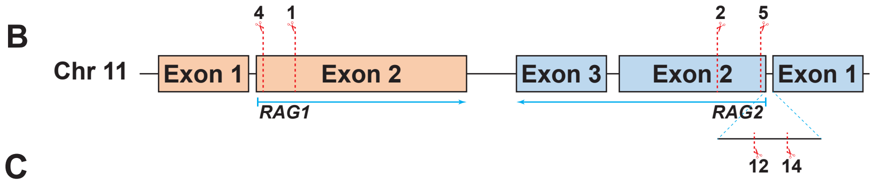

C

RAG1 (gRNA #)	gRNA sequence (PAM sequence bolded)	Specificity (*in silico* score)	Efficiency (*in silico* score)	INDEL % (*in vitro*, Plasmid)
1	TCTTTTCAAAGGATCTCACC**CGG**	98.29	51.06	6.20%
4	AACTGAGTCCCAAGGTGGGT**GGG**	87.91	65.83	21.95%

D

RAG2 (gRNA #)	gRNA sequence (PAM sequence bolded)	Specificity (*in silico* score)	Efficiency (*in silico* score)	INDEL % (*in vitro*, Plasmid)
2	CCAGATGGAAAACTCCAGTG**GGG**	60.77	75.26	27.05%
5	AAATTCATCAGTGAGAAGCC**TGG**	50.13	58.51	27.70%
12	CCTAGGGAGCATATAGTCCG**TGG**	91.40	59.80	52.45%
14	TGTCGGGCAAACCGCTACCC**TGG**	94.70	58.00	38.95%

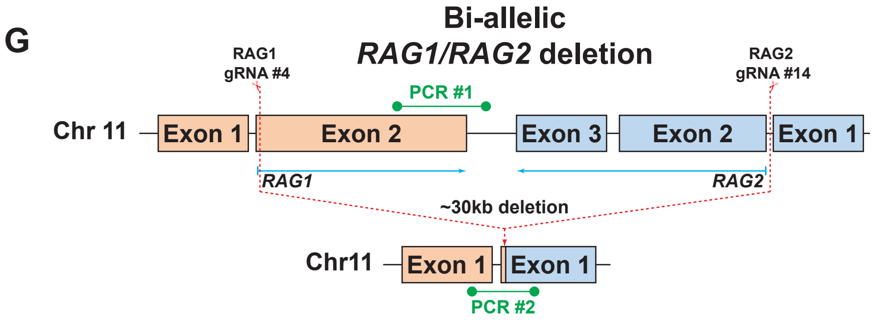

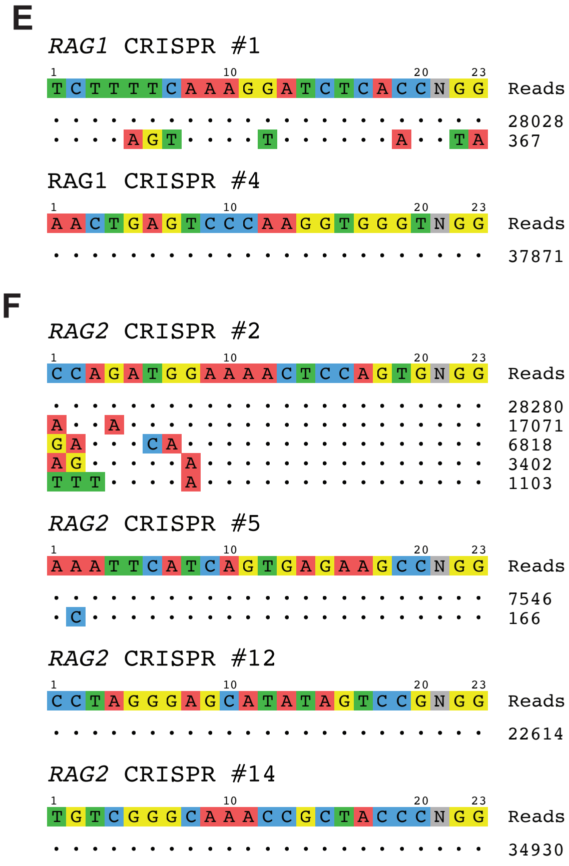

Supplementary Fig. 1 | Strategy for CRISPR/Cas9 gene excision to generate *RAG1*/*RAG2* double knockout PSCs.

(a) Schematic of the screening process for selecting gRNAs for each gene, *RAG1* and *RAG2,* with high on-target and low-off target activity *in vitro*.

(b) Schematic of the region on Chromosome 11 containing the coding sequences of *RAG1* and *RAG2*. gRNAs were designed to target sites around the start of the coding sequences, located in exon 2, for both genes.

(c-d) Summary of gRNA and PAM (bolded) sequences and predicted on- and off-target scores for gRNAs targeting *RAG1* **(c)** and *RAG2* **(d)** *in silico* (scored on a scale of 100). On-target activity in K562 cells is shown as the percentages of insertion deletion mutations (INDEL %) calculated via Sanger Sequencing (TIDE).

(e-f) Off-target gRNA activity analysis via GUIDE-seq. On-target reads are represented by black dots, and off-target reads are denoted by their mismatched bases.

(g) Design of the gene ablation strategy of the *RAG1-RAG2* locus in PSCs using *RAG1* gRNA #4 and *RAG2* gRNA #14. PCR primers, as shown, were designed to detect bi-allelic deletion of *RAG1* and *RAG2*.

Supplementary Figure 2

A

PSC → EMO → ATO

Mesoderm Induction			Hematopoietic Induction		T cell differentiation
Day -17	Day -16	Day -15	Day -14 to -7	Day -6 to 0	Week 0 to Week 7
XVIVO15	XVIVO15	XVIVO15	EGM2	EGM2	RB27 Media
rhBMP4	rhBMP4	rhBMP4	SB Blocker	SB Blocker	Ascorbic Acid
rhVEGF	rhVEGF	rhVEGF		rhSCF	rhSCF
rhFGF	rhFGF	rhFGF		rhFLT3L	rhFLT3L
rhActivinA				rhTPO	rh-IL7

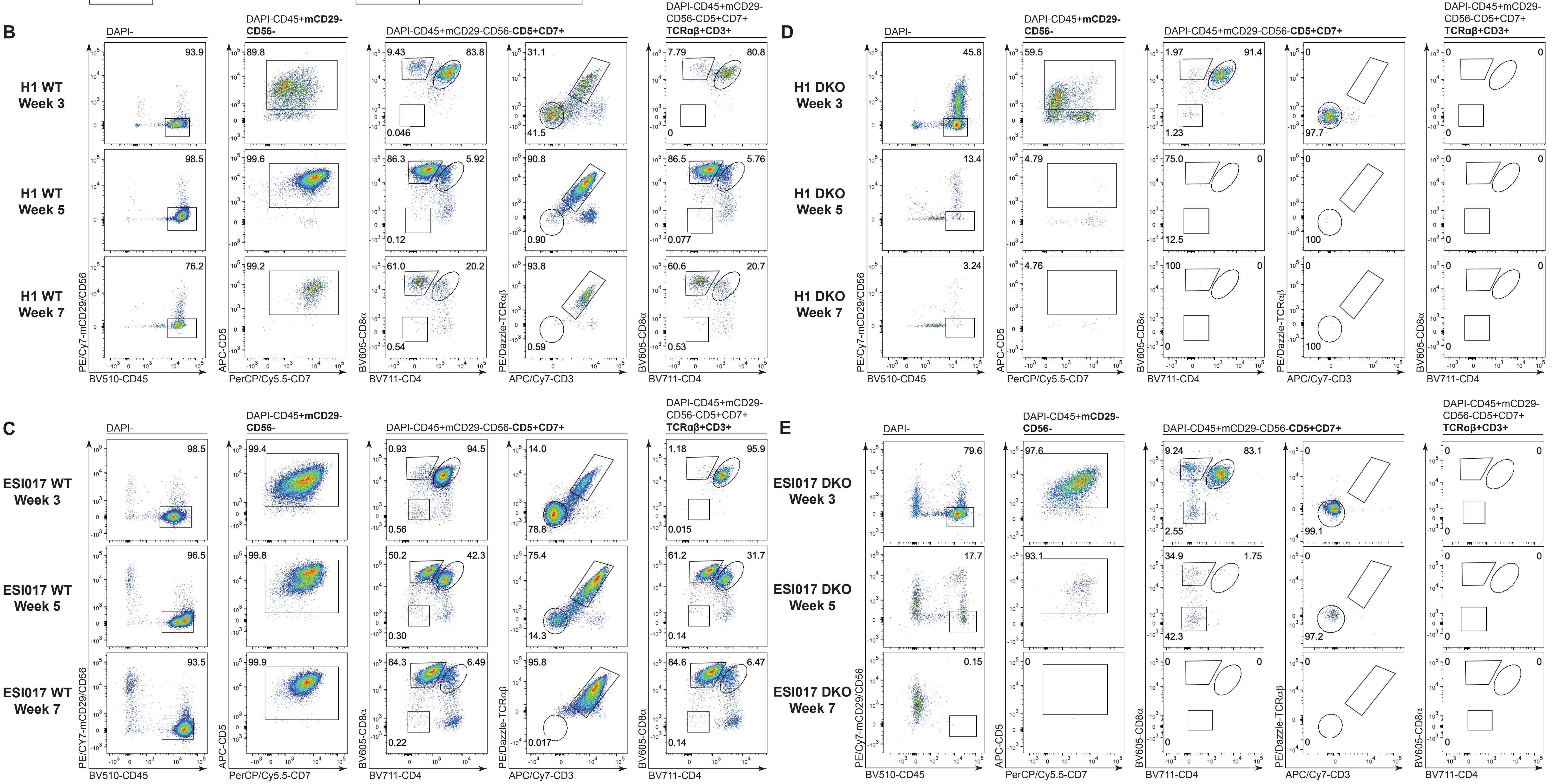

Supplementary Fig. 2 | Differentiation kinetics of WT and DKO PSCs in the ATO system.
(a) Schematic of the protocol used to generate T cells from PSCs in ATOs. After 3.5 days of mesoderm induction (days -17 to -15), hEMPs were harvested and aggregated with MS5-hDLL4 in embryonic mesodermal organoids (EMOs) for 2 weeks in hematopoietic induction media (days -14 to 0). At day 1, the artificial thymic organoid (ATO) phase, T cell differentiation was initiated by changing to T cell differentiation medium.
(b-e) Representative flow cytometry plots of the gating strategy used to track the progress of T cell differentiation from H1 WT PSCs **(b)**, ESI017 WT PSCs **(c)**, H1 DKO PSCs **(d),** and ESI017 DKO PSCs **(e)** in the ATO system at the indicated time points. Gating strategy is indicated above panels, and numbers indicate percentage of cells within each gate.

Supplementary Figure 3

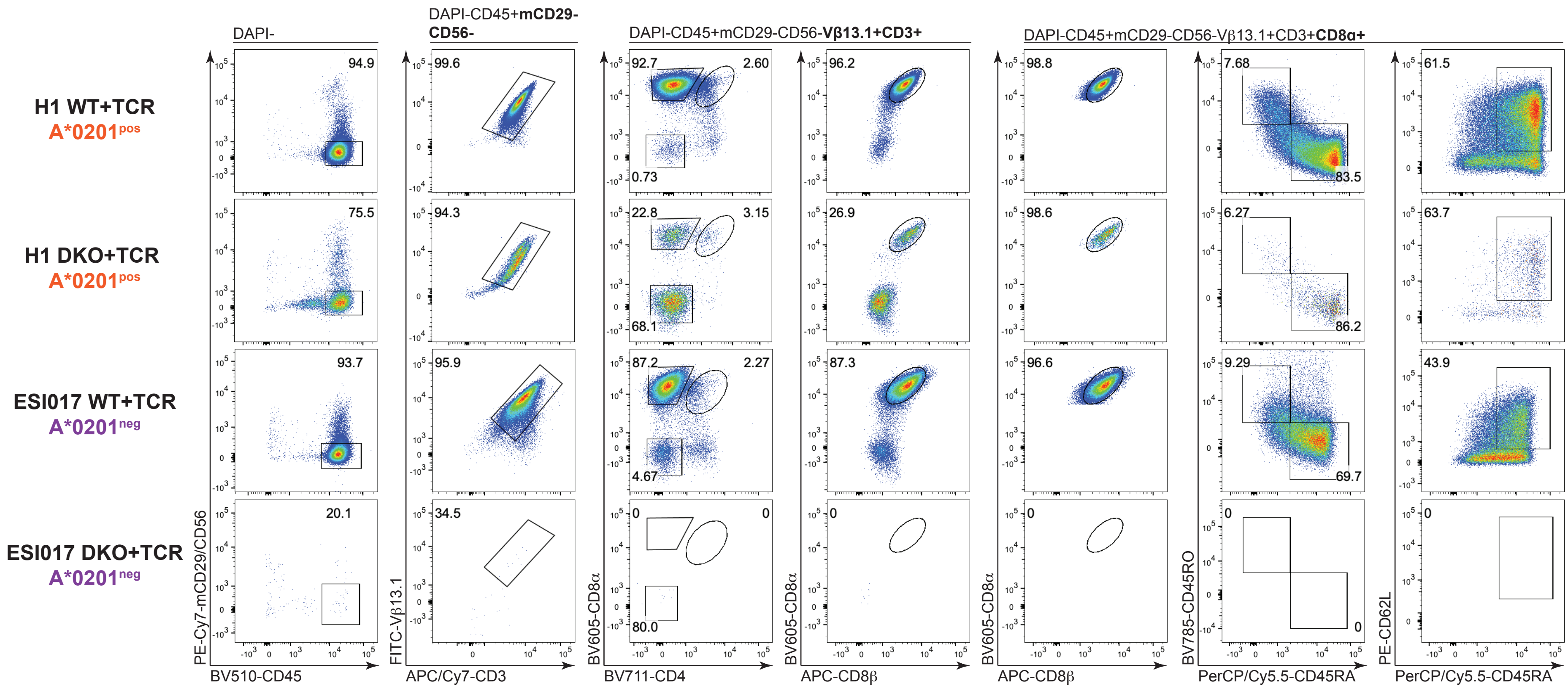

Supplementary Fig. 3 | Analysis of mature, conventional SP8 T cells from PSCs transduced with 1G4 TCR in the ATO system.
WT and *RAG1/RAG2* DKO PSCs from both PSC lines, H1 (A*0201^{pos}) and ESI017 (A*0201^{neg}) were transduced to express the HLA-A*0201-restricted 1G4 TCR recognizing the NYESO157-165 peptide, generating the WT+TCR and DKO+TCR lines. Representative flow cytometry analysis for the gating strategy used to identify maturation markers for conventional T cells from WT+TCR and DKO+TCR ATOs, harvested after 7 weeks of T cell differentiation (**main figures 2a-c).** Numbers indicate percentage of cells within each gate.

Supplementary Figure 4

A

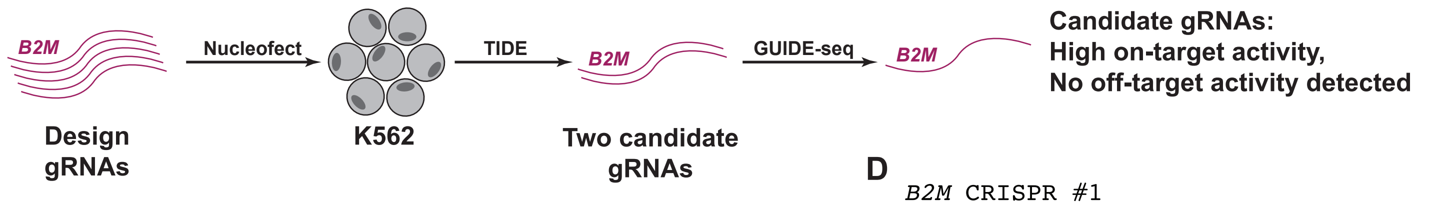

B

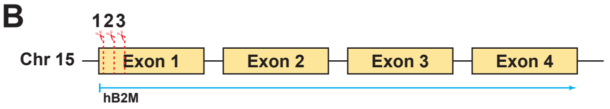

C

B2M (gRNA #)	**gRNA sequence** (PAM sequence bolded)	**Specificity** (*in silico* score)	**Efficiency** (*in silico* score)	**INDEL %** (*in vitro*, Plasmid)
**1**	TATAAGTGGAGGCGTCGCGC**TGG**	**98.29**	**51.06**	**25.30%**
**2**	GGCCGAGATGTCTCGCTCCG**TGG**	**94.06**	**59.37**	**57.35%**
**3**	GAGTAGCGCGAGCACAGCTA**AGG**	**89.51**	**56.28**	**45.40%**

D

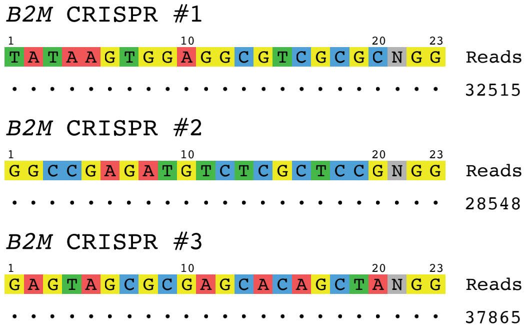

E

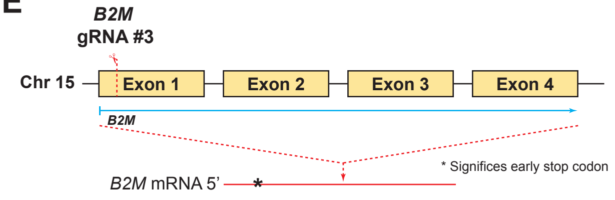

F

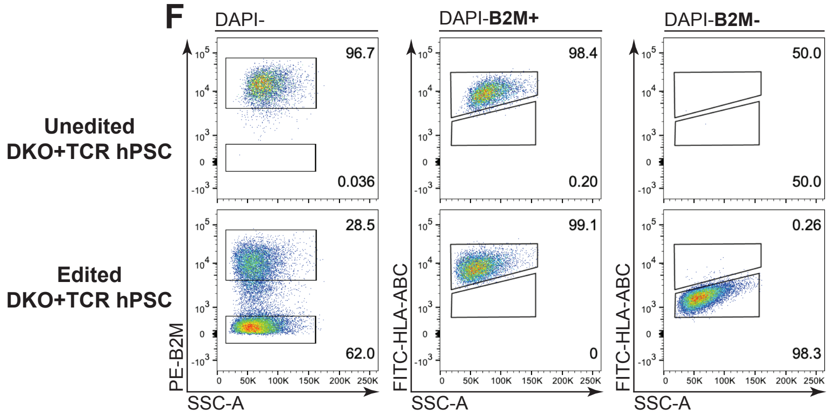

Supplementary Fig. 4 | Generation of polyclonal *B2M* knockout PSCs by INDEL.
(a) Schematic of the screening process for selecting gRNA for *B2M* with high on-target and low-off target activity in vitro.
(b) Schematic of the region on Chromosome 15 containing the coding sequence of *B2M* where gRNAs were designed to target sites near the start of the coding sequence in Exon 1.
(c) Summary of gRNA and PAM (bolded) sequences and predicted on- and off-target scores for gRNAs targeting *B2M*. On-target activity in K562 cells is shown as the percentage of insertion deletion mutations (INDEL %) calculated via Sanger Sequencing (TIDE).
(d) Off-target gRNA activity analysis via the GUIDE-seq method. On-target reads are represented by black dots, and off-target reads are denoted by their mismatched bases.
(e) Design of the gene knockout strategy of *B2M* in PSCs using *B2M* gRNA #3.
(f) Isolation of *RAG1*/*RAG2*-null (DKO)+TCR PSCs with successful *B2M* knockout by flow cytometry. Phenotype of knockout PSCs shown with HLA-A,B,C and *B2M* staining.

Supplementary Figure 5

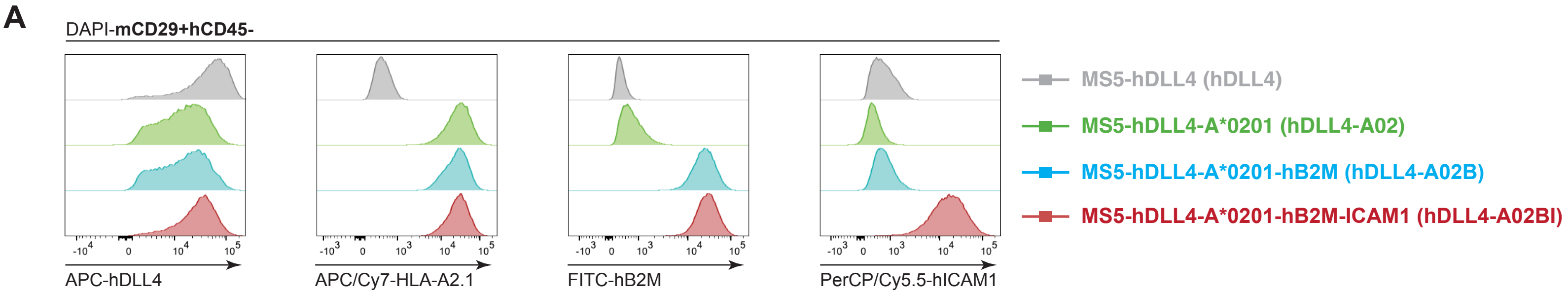

Supplementary Fig. 5 | Expression of human transgenic genes in engineered MS5 stromal cell lines

Representative flow cytometry histograms measuring the expression of *hDLL4*, HLA-A2.1, *hB2M*, and *ICAM1* on the transduced and FACS sorted MS5 stromal cell lines in the current study: MS5-hDLL4 (hDLL4), MS5-hDLL4-A*0201 (hDLL4-A02), MS5-hDLL4-A*0201-hB2M (hDLL4-A02B), MS5-hDLL4-A*0201-hB2M-ICAM1 (hDLL4-A02BI)

Supplementary Figure 6

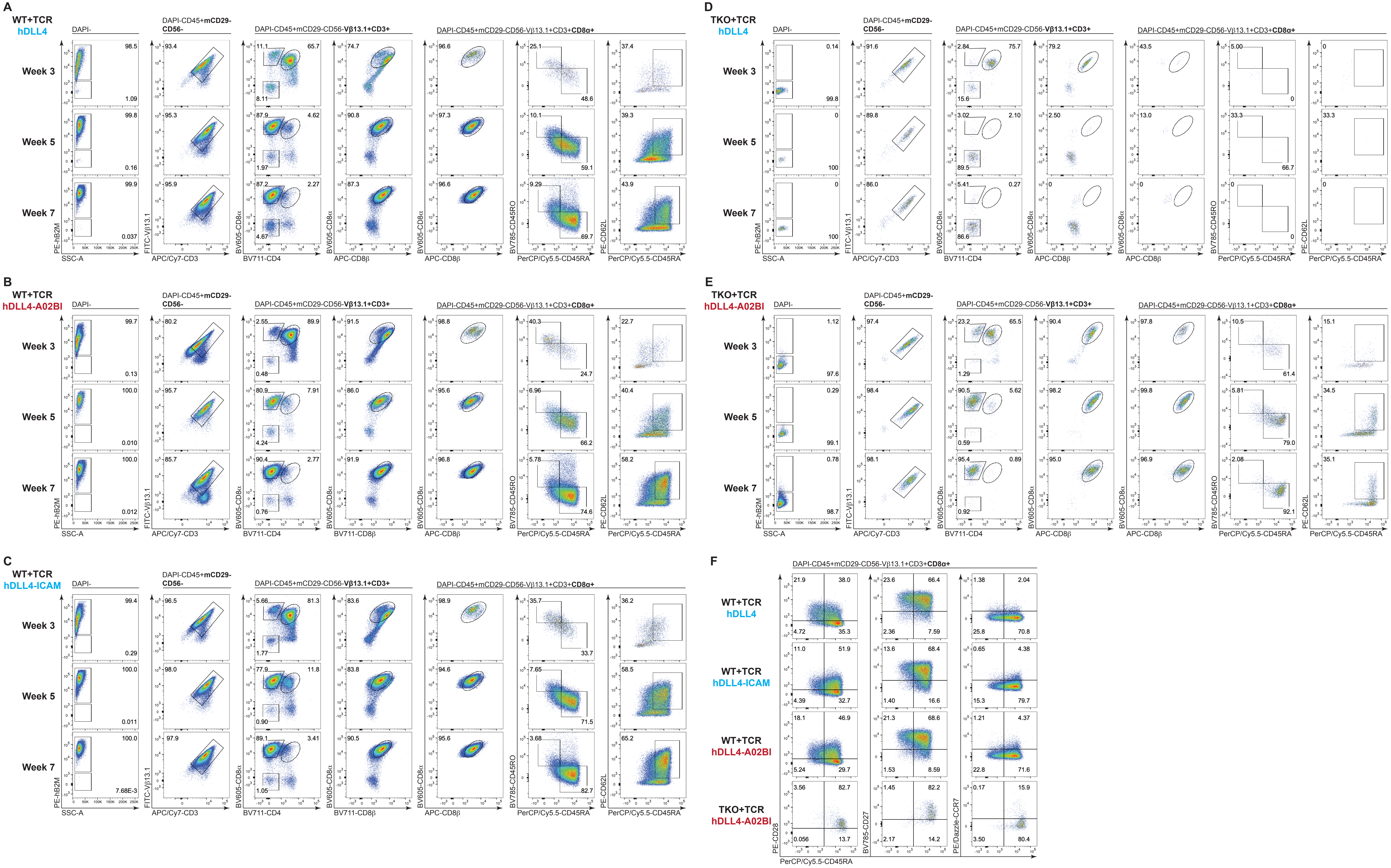

Supplementary Fig. 6 | Differentiation kinetics of WT+TCR and TKO+TCR PSCs with MS5-hDLL4 stroma cell line derivatives

(a-e) Representative flow cytometry plots of the gating strategy used to track the progress of T cell differentiation from ESI017 WT+TCR PSCs with hDLL4 stroma **(a)**, hDLL4-A02BI stroma **(b)**, and hDLL4-ICAM stroma **(c)**, and ESI017 TKO+TCR PSCs with hDLL4 stroma **(d)** or hDLL4-A02BI stroma **(e).** Gating strategy is indicated above panels, and numbers indicate percentage of cells within each gate.

(f) Flow cytometry plots showing staining for conventional, naïve T cell markers CD28, CD27, and CCR7 from Week 7 ATOs. Gating strategy is indicated above panels, and numbers indicate percentage of cells within each gate.

Supplementary Figure 7

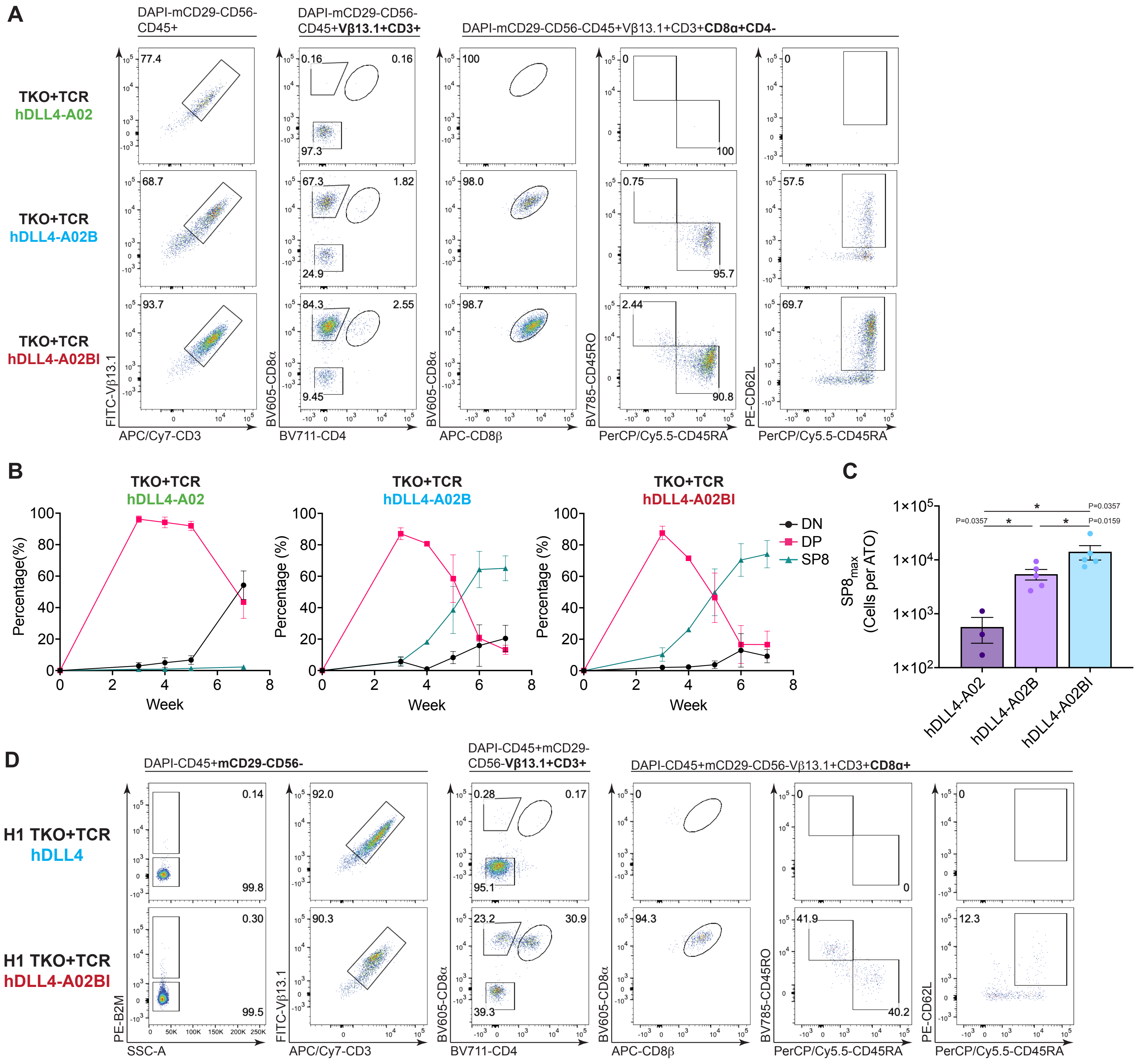

Supplementary Fig. 7 | Generation of Class I MHC-null, positively selected, antigen-specific SP8 T cells from TKO+TCR PSCs is dependent on hB2M transduction in MS5-hDLL4-A*0201 stromal cells, and significantly improved by the addition of ICAM1.

(a) Representative flow cytometry plots from ESI017 TKO PSCs transduced with the HLA-A*0201-restricted 1G4 TCR recognizing the $NYESO_{157-165}$ peptide (TKO+TCR), and differentiated 7 weeks in the ATO system with the following stromal conditions: hDLL4-A02, hDLL4-A02B, hDLL4-A02BI. After 7 weeks of T cell differentiation, TKO+TCR PSC-derived ATOs were analysed for maturation markers of conventional T cells. Gating strategy is indicated above panels, and numbers indicate percentage of cells within each gate.

(b) Frequency of T cell phenotypes (i.e., double negative (DN), double positive (DP), and SP8 T cells) over 7 weeks of differentiation with specialized stroma. Mean ± SEM (*$p<0.05$, two-tailed Mann-Whitney *U* test) are shown for each group (n=3 independent experiments for hDLL4-A02, and n=5 for both hDLL4-A02B and hDLL4-A02BI).

(c) Maximum SP8 T cell output per ATO reached over the 7-week course of T cell differentiation ($\mathbf{SP8}_{max}$). Mean ± SEM (*$p<0.05$, two-tailed Mann-Whitney U test) are shown for each stromal condition (n=3 individual experiments for hDLL4-A02, n=5 for both hDLL4-A02B and hDLL4-A02BI).

(d) Representative flow cytometry plots from H1 TKO PSCs transduced with the HLA-A*0201-restricted 1G4 TCR (H1 TKO+TCR), and differentiated 7 weeks in the ATO system with either hDLL4 or hDLL4-A02BI stroma. Gating strategy is indicated above panels, and numbers indicate percentage of cells within each gate.

Supplementary Figure 8

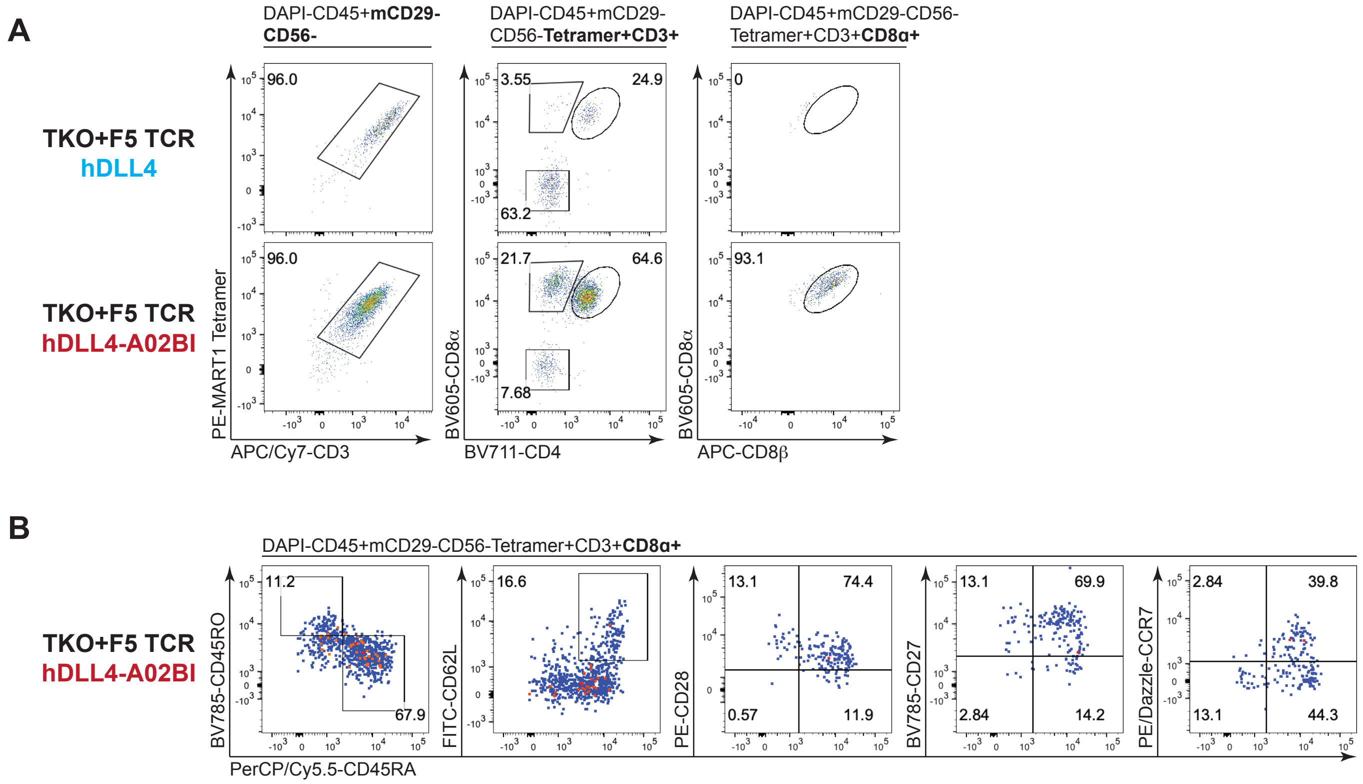

Supplementary Fig. 8 | ATOs generated with hDLL4-A02BI stromal cells facilitate positive selection of antigen-specific SP8 T cells from TKO PSCs transduced to express the alternative HLA-A*0201-restricted F5 TCR recognizing the MART1$_{26\text{-}35}$ peptide.

(a) Representative flow cytometry plots from ESI017 TKO PSCs transduced with the HLA-A*0201-restricted F5 TCR recognizing the MART1$_{26\text{-}35}$ peptide (TKO+F5 TCR), and differentiated 7 weeks in the ATO system with either hDLL4 or hDLL4-A02BI stroma. After 7 weeks of T cell differentiation, TKO+F5 TCR PSC-derived ATOs were analysed for maturation markers of conventional T cells (n=2 independent experiments). Gating strategy is indicated above panels, and numbers indicate percentage of cells within each gate.

(b) Flow cytometry plots showing staining for conventional, naïve T cell markers CD45RA, CD62L, CD28, CD27, and CCR7 from Week 7 ATOs initiated from TKO+F5 TCR PSCs. Gating strategy is indicated above panels, and numbers indicate percentage of cells within each gate.

Supplementary Figure 9

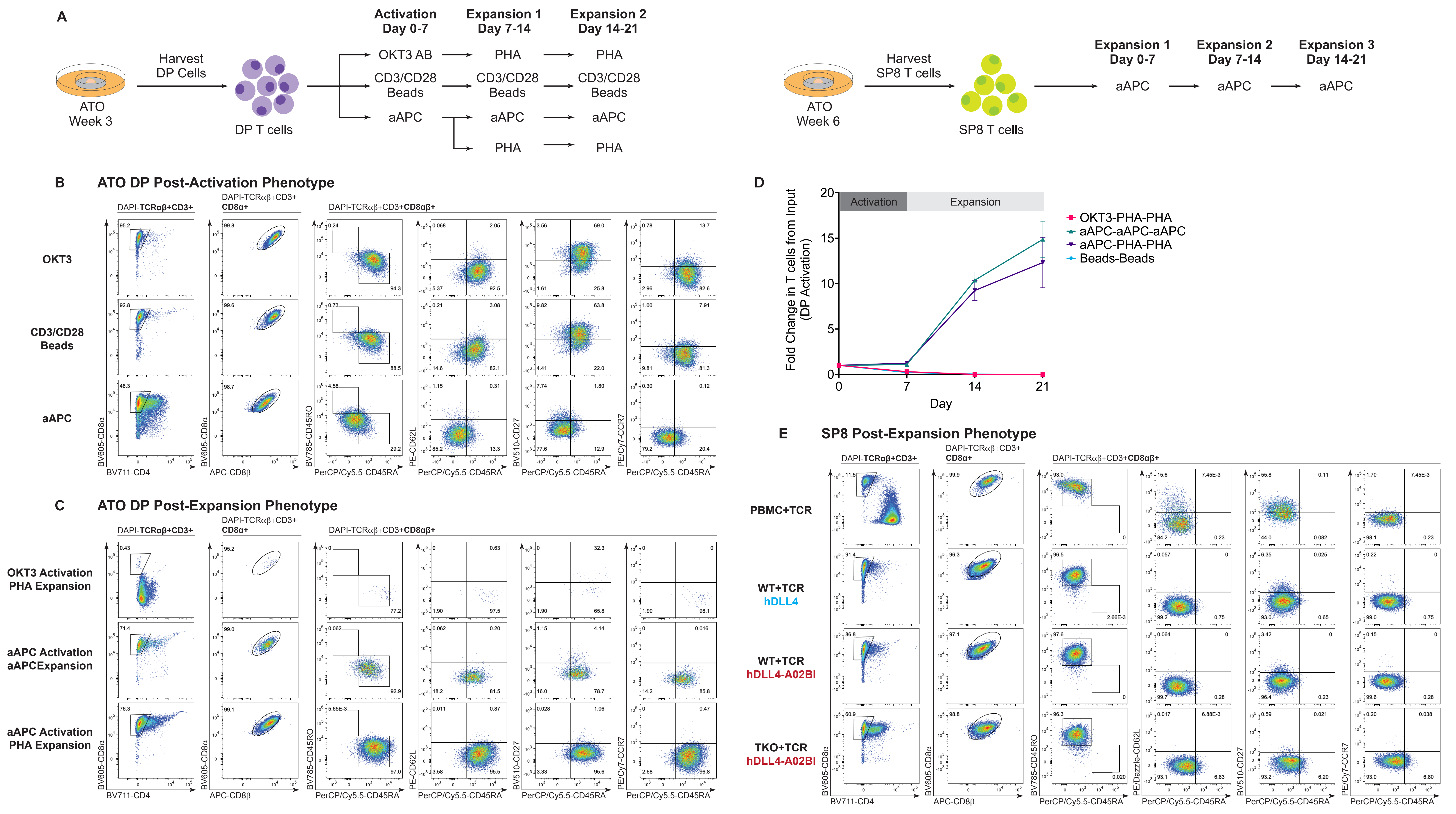

Supplementary Fig. 9 | Stimulation of ATO DP precursors induces an alternative SP8 phenotype after expansion, in comparison to expanded ATO SP8 T cells

(a) Schematic of the methods used to activate and expand DP precursors from Week 3 ATOs (left), and to expand SP8 T cells that were FACS isolated from mature, Week 6 ATOs (right).

(b-c) Representative flow cytometry plots tracking the phenotype and enrichment of SP8 T cells generated by various methods of activating DP T cells harvested from the ATO after 7 days **(b)**, and then the resulting phenotype of SP8 T cells after an additional 14 days of expansion (21 days total) **(c)**. ATO phenotype can be seen in Figure 3B and Supplementary Fig. 6C. Gating strategy is indicated above panels, and numbers indicate percentage of cells within each gate.

(d) Fold expansion of TKO+TCR DP T precursors immediately isolated from Week 3 ATOs in response to the specified methods (mean ± SEM shown; data are representative of 3 independent TKO+TCR PSC lines). Fold expansion of SP8 T cells from initial input of DP T cells are shown**.**

(e) Representative flow cytometry plots tracking the phenotype of SP8 T cells FACS-sorted directly from Week 6 ATOs and 3 cycles of expansion with aAPCs (21 days total). Top row shows sorted PBMCs transduced to express the 1G4 TCR. PBMC SP8 and PSC ATO-derived SP8s (both WT+TCR and TKO+TCR) maintain an effector-memory T cell phenotype ($CD8\alpha\beta^{+}CD4^{-}CD45RO^{+}CD45RA^{-}CD62L^{-}CD27^{-}CCR7^{-}$) after expansion. Gating strategy is indicated above panels, and numbers indicate percentage of cells within each gate.

Supplementary Figure 10

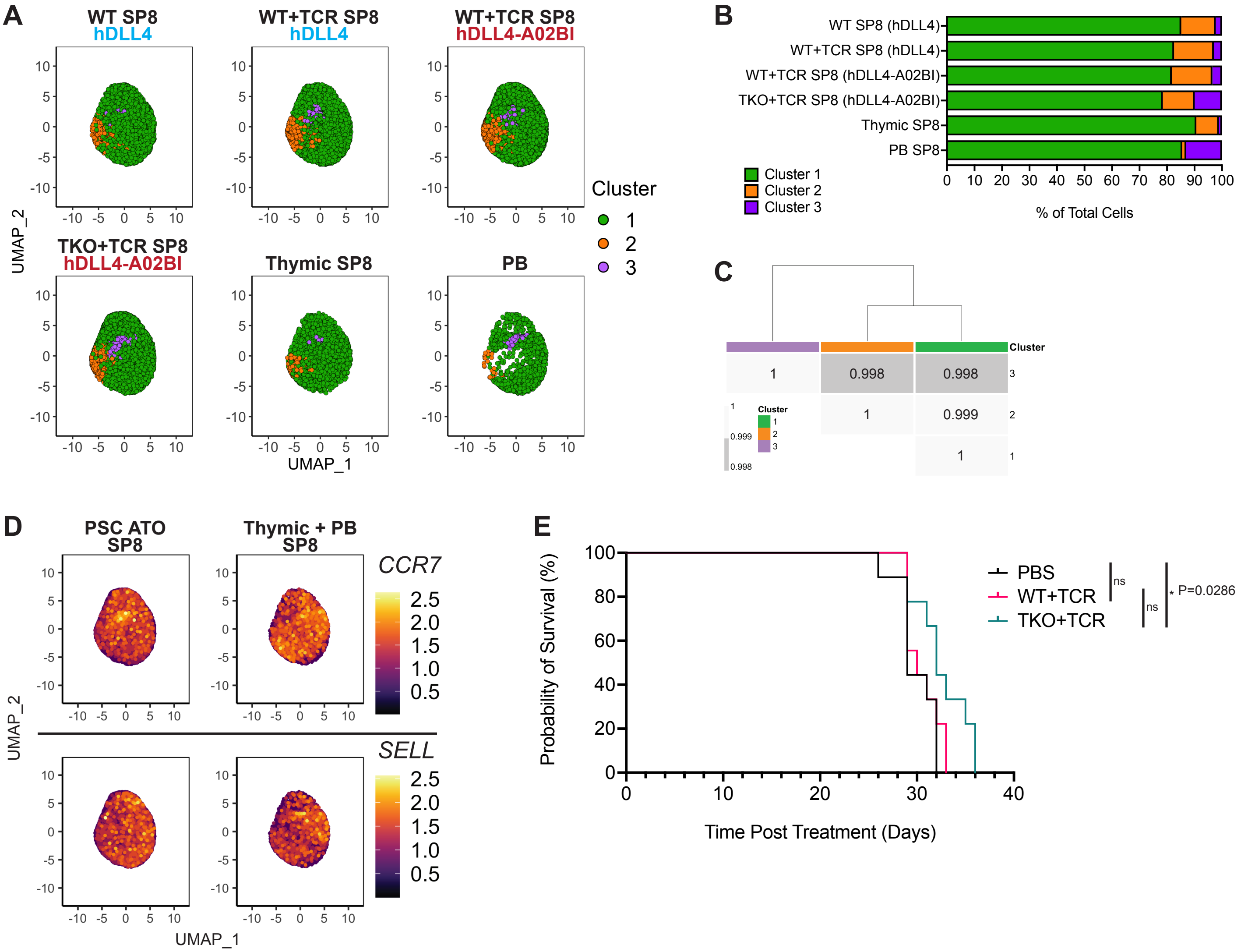

Supplementary Fig. 10 | Transcriptional and *in vivo* functional characterization of ATO SP8 T cells
(a) Uniform manifold approximation and projection (UMAP) visualization after SCTransform normalization and RPCA integration of ATO-derived SP8 T cells from WT PSCs with hDLL4 stroma (n=2, 4710 cells), WT+TCR PSCs with hDLL4 (n=2, 8618 cells) and hDLL4-A02BI (n=3, 12797 cells) stroma, and TKO+TCR PSCs with hDLL4-A02BI stroma (n=3, 7930 cells) in comparison with Thymic SP8 (n=2, 4793 cells), PB SP8 (n=1, 2083 cells). Principal components (PCs) and clusters were identified using the IKAP algorithm. **Note:** PB NK and monocytes have been removed from UMAP analysis shown in **main Figure 5A.**
(b) Frequency of clusters present in each SP8 population from *in vitro* and *in vivo* sources.
(c) Dendrogram of hierarchical clustering analysis and heatmap displaying Pearson's correlation of global gene expression for all pairwise combinations between each cluster (clusters shown horizontally in colours as per key and listed vertically to the right).
(d) UMAP-based visualization of *CCR7* (top) and *SELL* (bottom) expression in SP8s derived from all ATOs aggregated with PSCs (left) i.e. pooled data from WT PSCs with hDLL4 stroma (n=2, 4710 cells), WT+TCR PSCs with hDLL4 (n=2, 8618 cells) and hDLL4-A02BI (n=3, 12797 cells) stroma, and TKO+TCR PSCs with hDLL4-A02BI stroma (n=3, 7930 cells); and SP8s isolated from Thymus (n=2, 4793 cells) and Peripheral Blood (PB, n=1, 2083 cells) (right).
(e) Kaplan-Meier analysis of overall survival of NSG mice from *in vivo* tumour challenge (*$p<0.05$, log-rank test) from experiments shown in **main figure 8**. Briefly, mice were intravenously (I.V.) engrafted with NALM6 tumour cells (5×10^5 per mouse) expressing the cognate NYESO single chain trimer and firefly luciferase. 5 days after tumour engraftment, mice were injected I.V. with PBS, WT+TCR SP8 T cells (1×10^7 per mouse), or TKO+TCR SP8 T cells (1×10^7 per mouse). Survival curves were defined based on either spontaneous death or euthanized upon distress and discomfort as per animal care guidelines.
