# Supplementary material for: Generation of antigen-specific mature T cells from RAG1−/−RAG2−/−B2M−/− stem cells by engineering their microenvironment
Source: Nat Biomed Eng. 2023 Dec 7;8(4):461–78. doi: 10.1038/s41551-023-01146-7 (PMC11087257; doi:10.1038/s41551-023-01146-7)
Supplement: Supplementary file 2 [file 41551_2023_1146_MOESM2_ESM.pdf]

nature portfolio

Corresponding author(s): Gay M. Crooks

Last updated by author(s): Oct 10, 2023

# Reporting Summary

Nature Portfolio wishes to improve the reproducibility of the work that we publish. This form provides structure for consistency and transparency in reporting. For further information on Nature Portfolio policies, see our Editorial Policies and the Editorial Policy Checklist.

## Statistics

For all statistical analyses, confirm that the following items are present in the figure legend, table legend, main text, or Methods section.

| n/a | Confirmed | |
|---|---|---|
| ☐ | ☒ | The exact sample size (*n*) for each experimental group/condition, given as a discrete number and unit of measurement |
| ☐ | ☒ | A statement on whether measurements were taken from distinct samples or whether the same sample was measured repeatedly |
| ☐ | ☒ | The statistical test(s) used AND whether they are one- or two-sided<br>*Only common tests should be described solely by name; describe more complex techniques in the Methods section.* |
| ☒ | ☐ | A description of all covariates tested |
| ☒ | ☐ | A description of any assumptions or corrections, such as tests of normality and adjustment for multiple comparisons |
| ☐ | ☒ | A full description of the statistical parameters including central tendency (e.g. means) or other basic estimates (e.g. regression coefficient) AND variation (e.g. standard deviation) or associated estimates of uncertainty (e.g. confidence intervals) |
| ☐ | ☒ | For null hypothesis testing, the test statistic (e.g. *F*, *t*, *r*) with confidence intervals, effect sizes, degrees of freedom and *P* value noted<br>*Give P values as exact values whenever suitable.* |
| ☒ | ☐ | For Bayesian analysis, information on the choice of priors and Markov chain Monte Carlo settings |
| ☐ | ☒ | For hierarchical and complex designs, identification of the appropriate level for tests and full reporting of outcomes |
| ☐ | ☒ | Estimates of effect sizes (e.g. Cohen's *d*, Pearson's *r*), indicating how they were calculated |

*Our web collection on statistics for biologists contains articles on many of the points above.*

## Software and code

Policy information about availability of computer code

| | |
|---|---|
| Data collection | BDFACSDiva software v8.0.2 was used for flow cytometry data collection.<br>Living Image version 4.7.4 was used for the measurement and analysis of in vivo luciferase activity. |
| Data analysis | FlowJo v10.8.1 was used for the analysis of flow-cytometry data.<br>Benchling was used for sgRNA and vector designs (www.benchling.com).<br>GUIDEseq v1.0.1 was used for off-target sgRNA analysis (Tsai Lab).<br>GraphPad Prism v9.4.1 was used for graphical representations and statistical analysis.<br>CellRanger v7.0.0 from 10X Genomics was used for aligning raw TCR-sequencing reads.<br>Seurat v4.3.0 from the Satija Lab was run on RStudio v2022.07.1+554 with R v4.2.1 were used for filtering, cleaning and identifying single cells from gene expression and TCR sequencing data from CellRanger outputs. The codes are available on request.<br>DoubletFinder v2.0.3 from McGinnis et al. 2019, for identifying doublets, bioinformatically.<br>IKAP from Chen et al. 2019, for unsupervised clustering of ATO-derived T cells.<br>SingleCellExperiment from Amezquita et al. 2020, for extracting counts for pseudobulk analysis.<br>DEseq2 from Love et al. 2014, for performing normalizing pseudobulk data.<br>pheatmap from Kolde. R, 2019, for visualizing normalized Pearson's correlation data.<br>Living Image version 4.7.4 was used for the analysis of in vivo luciferase activity. |

For manuscripts utilizing custom algorithms or software that are central to the research but not yet described in published literature, software must be made available to editors and reviewers. We strongly encourage code deposition in a community repository (e.g. GitHub). See the Nature Portfolio guidelines for submitting code & software for further information.

# Data

Policy information about availability of data

All manuscripts must include a data availability statement. This statement should provide the following information, where applicable:

- Accession codes, unique identifiers, or web links for publicly available datasets
- A description of any restrictions on data availability
- For clinical datasets or third party data, please ensure that the statement adheres to our policy

The main data supporting the results in this study are available within the paper and its Supplementary information. Source data for the figures are provided with this paper. The raw and analysed datasets generated during this study for gene expression and TCR-encoding genes are available from the NCBI Gene Expression Omnibus (GSE237780). The public datasets used for this study can be found at the NCBI Gene Expression Omnibus for the human thymic SP8, GSE14898127, and at 10X Genomics28,29 (https://www.10xgenomics.com/resources/datasets/8-k-pbm-cs-from-a-healthy-donor-2-standard-2-1-0, https://www.10xgenomics.com/resources/datasets/human-pbmc-from-a-healthy-donor-10-k-cells-v-2-2-standard-5-0-0) for peripheral blood natural killer cells and CD14+ monocytes. The raw and analysed datasets generated during the study are available for research purposes from the corresponding author on reasonable request.

## Human research participants

Policy information about studies involving human research participants and Sex and Gender in Research.

| | |
|---|---|
| Reporting on sex and gender | The study did not involve human research participants. |
| Population characteristics | — |
| Recruitment | — |
| Ethics oversight | — |

Note that full information on the approval of the study protocol must also be provided in the manuscript.

# Field-specific reporting

Please select the one below that is the best fit for your research. If you are not sure, read the appropriate sections before making your selection.

☒ Life sciences ☐ Behavioural & social sciences ☐ Ecological, evolutionary & environmental sciences

For a reference copy of the document with all sections, see nature.com/documents/nr-reporting-summary-flat.pdf

# Life sciences study design

All studies must disclose on these points even when the disclosure is negative.

| | |
|---|---|
| Sample size | Sample sizes were determined via preliminary experiments, and by relying on similar published studies within the field (such as the below studies included in the reference list of the paper).<br><br>24. Stegen, S. J. C. van der et al. Generation of T-cell-receptor-negative CD8αβ-positive CAR T cells from T-cell-derived induced pluripotent stem cells. Nat. Biomed. Eng. 1–14 (2022) doi:10.1038/s41551-022-00915-0.<br>26. Wang, B. et al. Generation of hypoimmunogenic T cells from genetically engineered allogeneic human induced pluripotent stem cells. Nat. Biomed. Eng. 5, 429–440 (2021). |
| Data exclusions | No data were excluded. |
| Replication | The xperimental findings were reproduced in biological and/or technical replications, as indicated in the figure legends. |
| Randomization | Animals were assigned into each group on the basis of average luciferase-signal activity 5 days after tumour engraftment. The assignment criteria were to achieve relative equal average tumour burden in each group; then all groups were randomly selected as either experiment or control. |
| Blinding | For the in vivo experiments, mice were injected by an operator who was blinded to the treatment groups. All groups were treated with the exact same procedures and experienced the same imaging sessions. The imaging signals were batch-generated by imaging software; therefore, there was no bias for either the experimental group or the control group. Blinding was not used for the in vitro experiments, as each cell line used had distinctive phenotypes. |

# Reporting for specific materials, systems and methods

We require information from authors about some types of materials, experimental systems and methods used in many studies. Here, indicate whether each material, system or method listed is relevant to your study. If you are not sure if a list item applies to your research, read the appropriate section before selecting a response.

## Materials & experimental systems

| n/a | Involved in the study |
|---|---|
| ☐ | ☒ Antibodies |
| ☐ | ☒ Eukaryotic cell lines |
| ☒ | ☐ Palaeontology and archaeology |
| ☐ | ☒ Animals and other organisms |
| ☒ | ☐ Clinical data |
| ☒ | ☐ Dual use research of concern |

## Methods

| n/a | Involved in the study |
|---|---|
| ☒ | ☐ ChIP-seq |
| ☐ | ☒ Flow cytometry |
| ☒ | ☐ MRI-based neuroimaging |

## Antibodies

Antibodies used

Antibody List [company, (clone), RRID, catalogue number, dilution]:
Anti-human Vβ13.1 - FITC [Biolegend (clone H131) RRID: AB_2564032, 362404, 1:20]
Anti-human B2M - FITC [Biolegend (clone 2M2) RRID: AB_492837, 316304, 1:20]
Anti-human CD62L - PE [Biolegend (clone DREG-56) RRID: AB_314466, 304806, 1:20]
Anti-human CD62L - FITC [Biolegend (clone DREG-56) RRID: AB_314464, 304804, 1:20]
Anti-human CD28 - PE [Biolegend (clone CD28.2) RRID: AB_314310, 302908, 1:20]
Anti-human CD5 - APC [Biolegend (clone UCHT2) RRID: AB_314098, 300612, 1:20]
Anti-human CD8β - APC [Miltenyi (clone REA715) RRID: AB_2659522, 130-110-511, 1:20]
Anti-human DLL4 - APC [Biolegend (clone MHD4-46) RRID: AB_11204071, 346508, 1:20]
Anti-human CD107α - APC [Biolegend (clone H4A3) RRID: AB_1279055, 328620, 1:20]
Anti-human CD137 - APC [Biolegend (clone 4B4-1) RRID: AB_830672, 309810, 1:20]
Anti-human CD56 - PE/Cy7 [Biolegend (clone HCD56) RRID: AB_2563927, 362510, 1:20]
Anti-human CCR7 - PE/Cy7 [Biolegend (clone G043H7) RRID: AB_11126145, 353226, 1:20]
Anti-human CCR7 - PE/Dazzle [Biolegend (clone G043H7) RRID: AB_10913813, 353204, 1:20]
Anti-human CD25 - PE/Cy7 [Biolegend (clone BC96) RRID: AB_314282 302612 1:20]
Anti-human CD7 - PerCP/Cy5.5 [Biolegend (clone CD7-6B7) RRID: AB_2632912, 343116, 1:20]
Anti-human CD45RA - PerCP/Cy5.5 [Biolegend (clone HI100) RRID: AB_893357, 304122, 1:20]
Anti-human CD54 (ICAM1) - PerCP/Cy5.5 [Biolegend (clone HA58) RRID: AB_2715948, 353120, 1:20]
Anti-human TCRαβ - PE/Dazzle 594 [Biolegend (clone IP26) RRID: AB_2566599, 306726, 1:20]
Anti-human CD27 - BV510 [Biolegend (clone O323) RRID: AB_2562086, 302836, 1:20]
Anti-human CD27 - BV785 [Biolegend (clone O323) RRID: AB_11219185, 302831, 1:20]
Anti-human CD45 - BV510 Biolegend (clone HI30) RRID: AB_2561940, 304036, 1:20]
Anti-human CD56 - BV510 [Biolegend (clone HCD56) RRID: AB_2561944, 318340, 1:20]
Anti-human CD8α - BV510 [Biolegend (clone RPA-T4) RRID: AB_2564624, 344732, 1:20]
Anti-human CD8α - BV605 [Biolegend (clone RPA-T4) RRID: AB_2564391, 300556, 1:20]
Anti-human IL-2 - BV605 [Biolegend (clone MQ1-17H12) RRID: AB_2563877, 500332, 1:20]
Anti-human CD4 - BV711 [Biolegend (clone SK1) RRID: AB_2565243, 344734, 1:20]
Anti-human HLA-A2 - APC/Cy7 [Biolegend (clone BB7.2) RRID: AB_2561568, 343310, 1:20]
Anti-human CD3 - APC/Cy7 [Biolegend (clone UCHT1) RRID: AB_830754, 300425, 1:20]
Anti-human CD3 - PerCP/Cy5.5 [Biolegend (clone UCHT1) RRID: AB_893299, 300430, 1:20]
Anti-human CD3 - BV785 [Biolegend (clone UCHT1) RRID: AB_2687178, 300472, 1:20 ]
Anti-human CD326 (EPCAM) - PerCP/Cy5.5 [Biolegend (Clone 9C4) RRID: AB_2098808, 324214, 1:20]
Anti-human CD45RO - BV785 [Biolegend (clone UCHL1) RRID: AB_2563819, 304234, 1:20]
Anti-human HLA-A,B,C - FITC [Biolegend (clone W6/32) RRID: AB_314873, 311404, 1:20]
Anti-human IFNγ - BV785 [Biolegend (clone 4S.B3) RRID: AB_2563882, 502542, 1:20]
Anti-human TNFα - APC/Cy7 Biolegend (clone Mab11) RRID: AB_2562870, 502944, 1:20]
Anti-human CD137 - PE/Dazzle 594 [Biolegend (clone 4B4-1) RRID: AB_2566260, 309826, 1:20]
Anti-human CD3 Ultra-LEAF™ Purified Antibody [Biolegend (clone OKT3) RRID: AB_11150592, 317326, 500ng/mL]
Anti-mouse CD29 - PE/Cy7 [Biolegend (clone HMb1-1) RRID: AB_528790, 102222, 1:50]
iTAg Tetramer - HLA-A*02:01 NY-ESO-1 (SLLMWITQC) - PE [MBL International, TB-M011-1, 1:50]
iTAg Tetramer - HLA-A*0201 MART-1 (ELAGIGILTV) - PE [MBL International, TB-0009-1 1:50 ]

Validation

All antibodies used are commercially available and were validated for use in flow cytometry. Data are available on the manufacturer's website.

## Eukaryotic cell lines

Policy information about cell lines and Sex and Gender in Research

Cell line source(s)

MS5-hDLL4, K562-CD80-CD83-CD137L-HLA-A*0201/B2M/NYESO157-165 aAPC, K562-HLA-A*0201/B2M/NYESO(157-165)

| | |
|---|---|
| Cell line source(s) | aAPC, and K562-HLA-A*0201/B2M/MART1(26-35) aAPC were previously generated in the Crooks Lab. For this study, the MS5-hDLL4-ICAM, MS5-hDLL4-A*0201 (A02), MS5-hDLL4-A*0201-hB2M (A02B), and MS5-hDLL4-A*0201-hB2M-ICAM (A02BI) lines were generated in the Crooks Lab via transduction of the MS5-hDLL4 line with lentivirus.<br><br>The H1 and ESI017 PSC lines were purchased from WiCell and ESI BIO, respectively. ESI017 RAG1–/–RAG2–/– double knockout (DKO) and RAG1–/–RAG2–/–B2M–/– triple knockout (TKO) PSC lines; H1 DKO and TKO PSC lines; ESI017 DKO and TKO transduced with UBC-1G4TCR-mTagBFP2 (ESI017 DKO+TCR and ESI017 TKO+TCR) PSC lines; H1 DKO and TKO transduced with UBC-1G4TCR-mTagBFP2 (H1 DKO+TCR and H1 TKO+TCR) PSC lines; ESI017 and H1 transduced with UBC-1G4TCR-mTagBFP2 (ESI017 WT+TCR and H1 WT+TCR) PSC lines were generated for this study in the Crooks Lab.<br><br>293T cells were purchased from ATCC (Cat. CRL-3216) for the packaging of lentivirus particles. |
| Authentication | K562 aAPCs and MS5-hDLL4 lines were authenticated via flow cytometry for their respective markers. Certificates of authentication were provided for the PSC lines that were purchased from WiCell and ESI BIO. Edited PSC lines were authenticated by genotyping PCR, karyotyping, and flow cytometry. Changes in morphology and pluripotency were routeinly monitored via flow cytometry. |
| Mycoplasma contamination | The cell lines were routinely checked for mycoplasma contamination measured using bioluminesence; no infections were detected. |
| Commonly misidentified lines<br>(See ICLAC register) | No commonly misidentified cell lines were used. |

# Animals and other research organisms

Policy information about studies involving animals; ARRIVE guidelines recommended for reporting animal research, and Sex and Gender in Research

| | |
|---|---|
| Laboratory animals | We used NOD.Cg-Prkdcscid Il2rgtm1Wjl/SzJ (NSG) mice, purchased from The Jackson Laboratory (Strain #005557; RRID:IMSR_JAX:005557. Details can be found at https://www.jax.org/strain/005557). Female mice between the ages of 6–8 weeks were used. No further modification of the animals occurred before tumour engraftment and T cell treatment. and then bred at UCLA. 8–14-week-old female mice were used for the in vivo experiments.<br><br>All animals were housed in the UCLA-CNSI vivarium, which is maintained at approximately 20–26 °C, 30–70% humidity, with a 12-h-light/dark cycle (6am/6pm). Up to 5 NSG mice were kept in an individually ventilated cage. Details can be found at https://rsawa.research.ucla.edu/arc/temp-and-humidity. |
| Wild animals | The study did not use wild animals. |
| Reporting on sex | Sex was not considered in the study design, yet the findings apply to both sexes. |
| Field-collected samples | The study did not involve samples collected from the field. |
| Ethics oversight | All animal experiments were conducted under a protocol approved by the UCLA Chancellor's Animal Research Committee (ARC), also known as the UCLA institutional animal care and use committee (IACUC). |

Note that full information on the approval of the study protocol must also be provided in the manuscript.

# Flow Cytometry

## Plots

Confirm that:

☒ The axis labels state the marker and fluorochrome used (e.g. CD4-FITC).

☒ The axis scales are clearly visible. Include numbers along axes only for bottom left plot of group (a 'group' is an analysis of identical markers).

☒ All plots are contour plots with outliers or pseudocolor plots.

☒ A numerical value for number of cells or percentage (with statistics) is provided.

## Methodology

| | |
|---|---|
| Sample preparation | Cells were periodically harvested from filters over 7 weeks of in vitro T-cell differentiation. A total of 9 aggregates in 3 filters (3 aggregates/filter) were disassociated in MACS buffer (PBS, 0.5% BSA, 2mM EDTA), and filtered through a 50-µm strainer. Cells were blocked using FcX block (Biolegend) and stained with antibodies at a 1:20 dilution in a final volume of 100 µL with MACS buffer at 4 C. For intracellular cytokine assays, surface antibodies were stained prior to fixation, and fixation/permeabilization was performed according to the protocol provided by the BD fix/perm kit. |
| Instrument | Flow-cytometry samples were analysed using the BD LSRII Fortessa, and the samples were sorted using either the BD FACSARIA or FACSARIA-H instruments. |

| | |
|---|---|
| Software | BD systems used BDFACSDiva software v8.0.2 for flow-cytometry data collection.<br>FlowJo v10.8.1 was used for the analysis of flow-cytometry data. |
| Cell population abundance | Sorted populations, using BD either BD FACSARIA or FACSARIA-H instruments and Miltenyi MACS columns, were confirmed by flow cytometry. |
| Gating strategy | Flow-cytometry samples were first gated on cell size and granularity using FSC-A/SSC-A plots, drawing the lymphocyte gate. Doublets were excluded twice using FSC-H/SSC-H and FSC-W/SSC-W gates before dead cells were excluded using a viability stain [DAPI or Zombie (Biolegend)]. Gating strategies are shown in the supplementary information. |

☒ Tick this box to confirm that a figure exemplifying the gating strategy is provided in the Supplementary Information.
